# The alteration in the architecture of a T‐DNA insertion rice mutant *osmtd1* is caused by up‐regulation of *MicroRNA156f*

**DOI:** 10.1111/jipb.12340

**Published:** 2015-04-10

**Authors:** Qing Liu, Gezhi Shen, Keqin Peng, Zhigang Huang, Jianhua Tong, Mohammed Humayun Kabir, Jianhui Wang, Jingzhe Zhang, Genji Qin, Langtao Xiao

**Affiliations:** ^1^ Hunan Provincial Key Laboratory of Phytohormones and Growth Development Hunan Provincial Key Laboratory for Crop Germplasm Innovation and Utilization Hunan Agricultural University Changsha 410128 China; ^2^ Crop Institute Shanghai Academy of Agricultural Sciences Shanghai 201106 China; ^3^ Horticulture Institute Sichuan Academy of Agricultural Sciences Chengdu 610066 China; ^4^ State Key Laboratory of Protein and Plant Gene Research College of Life Sciences Peking University Beijing 100871 China

**Keywords:** *Oryza sativa*, *OsmiR156f*, plant architecture, protease inhibitor, T‐DNA insertion

## Abstract

Plant architecture is an important factor for crop production. Some members of *microRNA156* (*miR156*) and their target genes *SQUAMOSA* *Promoter‐Binding Protein‐Like* (*SPL*) were identified to play essential roles in the establishment of plant architecture. However, the roles and regulation of *miR156* is not well understood yet. Here, we identified a T‐DNA insertion mutant *Osmtd1* (*Oryza sativa* multi‐tillering and dwarf mutant). *Osmtd1* produced more tillers and displayed short stature phenotype. We determined that the dramatic morphological changes were caused by a single T‐DNA insertion in *Osmtd1*. Further analysis revealed that the T‐DNA insertion was located in the gene *Os08g34258* encoding a putative inhibitor I family protein. *Os08g34258* was knocked out and *OsmiR156f* was significantly upregulated in *Osmtd1*. Overexpression of *Os08g34258* in *Osmtd1* complemented the defects of the mutant architecture, while overexpression of *OsmiR156f* in wild‐type rice phenocopied *Osmtd1*. We showed that the expression of *OsSPL3*, *OsSPL12*, and *OsSPL14* were significantly downregulated in *Osmtd1* or *OsmiR156f* overexpressed lines, indicating that *OsSPL3*, *OsSPL12*, and *OsSPL14* were possibly direct target genes of *OsmiR156f*. Our results suggested that *OsmiR156f* controlled plant architecture by mediating plant stature and tiller outgrowth and may be regulated by an unknown protease inhibitor I family protein.

**Edited by:** Qian Qian, China National Rice Research Institute, China.

## INTRODUCTION

Plant architecture is important for plant morphology and crop production. Branching and plant height are key factors affecting plant architecture. As specialized branches bearing grains, rice tillers are formed on the un‐elongated basal internodes and grow independently from the mother culm with its own adventitious roots (Li 1979; Wang and Li 2005). Rice tillering is important for rice ideal plant architecture (IPA) which indicates a rice variety without unproductive tillers, with more grains per panicle, and with thick and sturdy stems. Because IPA plants have more potential for higher yields in rice (Jiao et al. 2010; Miura et al. 2010), it is feasible to increase rice yield by reducing the unproductive tillers in plant breeding.

MicroRNAs (miRNAs) are 21–24 nucleotide RNAs binding to target genes by imperfect base pairing and cause cleavage of target mRNA or repression of translation (Chen 2008). Being regarded as universal regulators of gene expression, many miRNAs have been proved to be essential in regulating plant architecture (Schwab et al. 2005; Xie et al. 2006; Chen 2008; Wang et al. 2011a; Fu et al. 2012; Wei et al. 2012). Among them, the *microRNA156* (*miR156*) family is a class of miRNAs playing pivotal roles in plant architecture. The *miR156* genes are conserved and highly expressed *miRNAs* in the plant kingdom. Computational and experimental analysis indicated that *miR156s* directly regulate the expression of *SQUAMOSA‐PROMOTER BINDING LIKE* (*SPL*) genes (Rhoades et al. 2002; Xie et al. 2006; Guo et al. 2008). *SPL* genes encode plant‐specific transcription factors containing a DNA‐binding motif highly conserved in SQUA promoter‐binding protein (SBP) (Shikata et al. 2009). The *miR156* and its target *SPL* genes are involved in various developmental processes. In *Arabidopsis*, *miR156* controls proper pattern formation during early embryogenesis by mediating *SPL10* and *SPL11* (Nodine and Barte 2010). The *miR156*, *miR172*, and *SPL* genes (including *SPL3/4/5*, *SPL9*, *SPL10*, *SPL15*) determine plant development by promoting juvenile/adult phase transition (Chuck et al. 2007; Wu et al. 2009; Jung et al. 2011; Willmann and Poethig 2011; Yang et al. 2011). The miR156‐targeted SPL factor *SPL9* affects leaf plastochron length and organ size (Wang et al. 2008) and regulates the anthocyanin accumulation during the transition from the leaf to flower formation (Gou et al. 2011). Enhanced *miR156b* expression leads to branching, altered trichome morphology, and increased seed carotenoid levels through the suppression of *SPL15* and other *SPL* target genes (Wei et al. 2012). In addition, it is also reported that miR156 is a phloem‐mobile signal regulating the process of tuberization in potato by targeting *StSPL3*, *StSPL6*, *StSPL9*, and *StSPL13* (Bhogale et al. 2014). In switchgrass, overexpression of *miR156* genes improves biomass by changing apical dominance and floral transition through suppressing target *SPL* genes (Chuck et al. 2011; Fu et al. 2012).

The rice genome contains 12 *miR156* genes, named *OsmiR156a* to *OsmiR156l*, and 19 *SPL* genes, named *OsSPL1* to *OsSPL19*, respectively. Except 11 *OsSPLs* (*OsSPL2*, *OsSPL3*, *OsSPL4*, *OsSPL7*, *OsSPL11*, *OsSPL12*, *OsSPL13*, *OsSPL14*, *OsSPL16*, *OsSPL17*, and *OsSPL18*), no other genes contained *OsmiR156* mature complementary sequences in rice genome, suggesting that *OsmiR156* specially targets *OsSPL* genes in rice (Xie et al. 2006). Among the 12 *miR156* genes in rice, 10 members (*OsmiR156a* to *OsmiR156j*) produce the same mature miRNA sequence, implying that a complex regulation network exists between *OsSPL* and the *OsmiR156* family. Indeed, previous studies have suggested that different *OsSPL* genes have a diverse tempo‐spatial expression patterns in rice. *OsSPL7*, *OsSPL12*, *OsSPL14*, *OsSPL16*, *OsSPL17*, and *OsSPL18* were predominantly expressed in young panicles; *OsSPL2*, *OsSPL4*, *OsSPL8*, *OsSPL10*, *OsSPL11*, and *OsSPL15* were highly expressed in stem, leaf sheath, and young panicles; while *OsSPL1*, *OsSPL3*, *OsSPL5*, *OsSPL6*, *OsSPL9*, and *OsSPL13* were expressed in all tissues. Overexpression *OsmiR156d* or *OsmiR156h* in rice resulted in decreased mRNA levels of three *OsSPL* genes (*OsSPL2*, *OsSPL12*, and *OsSPL13*) in the flag leaves, two *OsSPL* genes (*OsSPL16* and *OSPL18*) in the panicles, and one *OsSPL* gene (*OsSPL14*) in both flag leaves and panicles (Xie et al. 2006), suggesting that *OsSPL* genes may be tempo‐spatially regulated by *OsmiR156*.

Although *miR156s* have been elucidated to regulate diverse processes in plants, the function and regulation of *OsmiR156* family genes in rice remain elusive. In this paper, we identified a rice T‐DNA insertion mutant *Osmtd1* (*Oryza sativa* multi‐tillering and dwarf mutant). The *Osmtd1* produced many more tillers and reduced plant height. We located one T‐DNA insertion in *Os08g34258*, an unknown gene encoding a putative protease inhibitor, in *Osmtd1*. Overexpression of *Os08g34258* complemented the multi‐tillering and dwarf phenotypes of *Osmtd1*. We demonstrated that *OsmiR156f* was upregulated and *OsSPL3*, *OsSPL12*, and *OsSPL14* were downregulated in *Osmtd1*. Our findings provided a new layer of understanding on the function and regulation of *OsmiR156f* in rice architecture establishment.

## RESULTS

### The *Osmtd1* mutant exhibited dwarf and bushy phenotypes

**Figure 1:**
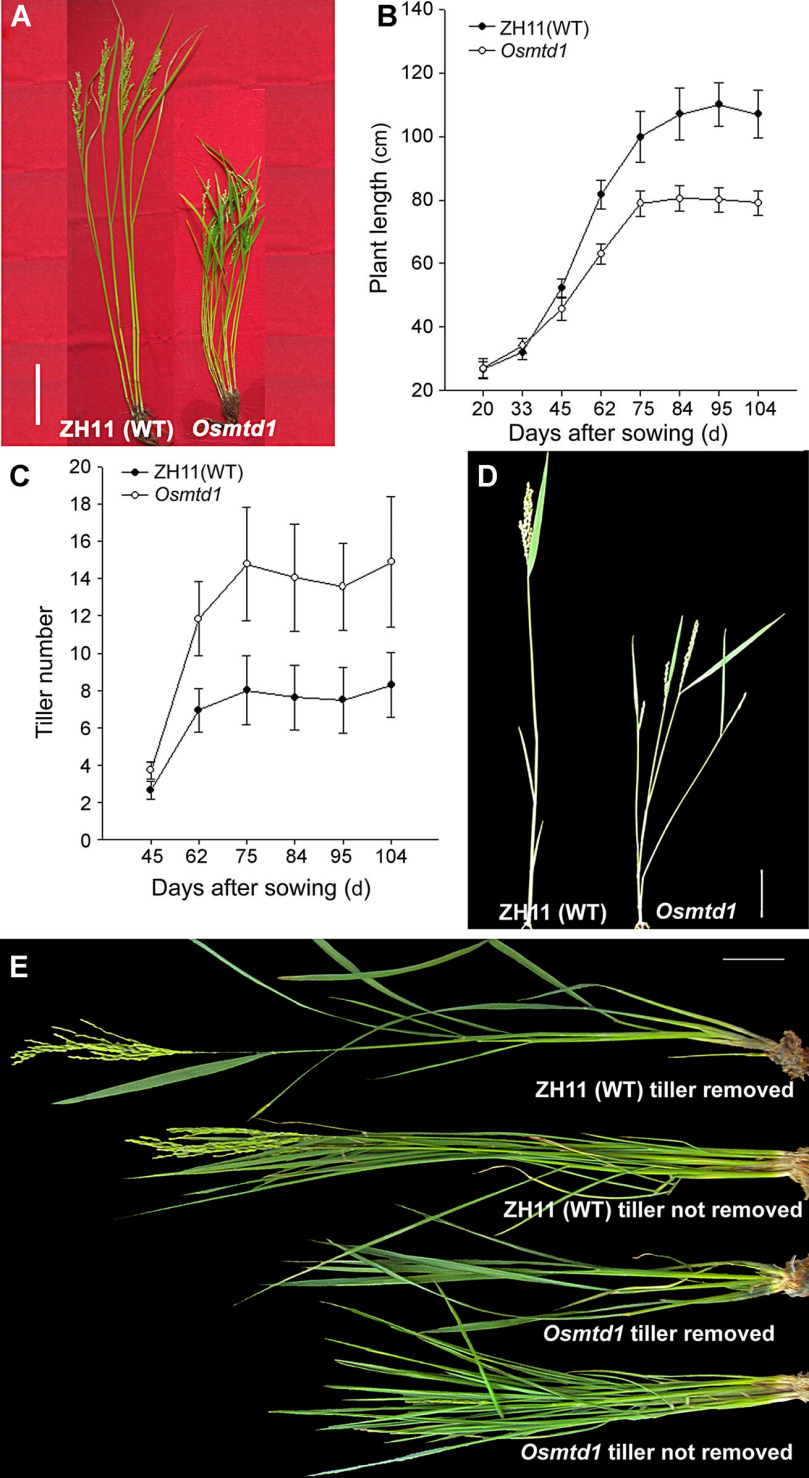
**The phenotypes of *Osmtd1*** (**A**) *Osmtd1* was significantly shorter and had more tillers than the wild‐type rice Zhonghua 11 (ZH11). (**B**) The plant height (cm) and (**C**) the tiller number are showed at different days after sowing. (**D**) The outgrowth of the higher node tiller in *Osmtd1*. (**E**) The influence of rice plant height after removing axillary tiller buds in *Osmtd1* and ZH11. Plant height of *Osmtd1* did not increase after removing the tillers while the wild‐type rice ZH11 did. Bar, 10 cm. All data and pictures were examined using the plants grown in the pots.

We identified a mutant from a rice T‐DNA insertion mutant collection and named it *Osmtd1* because the mutant displayed multi‐tillering and dwarfism phenotypes. Although no differences between *Osmtd1* and wild‐type plants were observed at the seedling stage, *Osmtd1* produced plant architecture distinct from wild‐type at the tillering stage. The phenotypes of *Osmtd1* include decreased plant height and increased tiller number (Figure 1A). To further illustrate *Osmtd1* phenotypes, we investigated the plant height and tiller number in a time course during plant development. *Osmtd1* was shorter than wild‐type rice from 45 to 104 d after sowing (DAS). At 75 DAS, the *Osmtd1* height was no longer increased, while the wild‐type rice further grew to a higher stature (Figure 1B). The analysis of tiller number indicated that there were no obvious differences between wild‐type rice and *Osmtd1* found at 45 DAS. However, *Osmtd1* produced more tillers than wild‐type rice after 62 DAS (Figure 1C). Usually, rice tillers are only formed on the un‐elongated basal internodes of rice main culm in wild‐type rice. However, we observed the outgrowth of tiller buds at the elongated higher internodes in *Osmtd1* (Figure 1D). In addition, the grain number was reduced and the primary panicle branches were shorter in *Osmtd1* (Figure 1D).

It has been suggested that dwarfism may be the secondary effect of the formation of more tillers, because removal of tillers rescues plant height in some rice mutants (Zou et al. 2006). To evaluate whether dwarf phenotype is caused by the excessive tillers in *Osmtd1*, we removed the new axillary tiller buds of wild‐type or *Osmtd1* every day from tillering stage to reproductive stage, and measured the plant height. The results showed that removal of tillers did not increase the height of *Osmtd1*, but did increase the height of wild‐type plants (Figure 1E), indicating that the dwarf trait of *Osmtd1* was independent from multi‐tillering and the mutation caused the high tillering and dwarfism simultaneously.

### The phenotypes of *Osmtd1* were caused by a single T‐DNA insertion

As *Osmtd1* was obtained from a T‐DNA mutant collection, we first test whether the phenotypes of *Osmtd1* could be resulted from T‐DNA insertion. We crossed *Osmtd1* with the wild‐type rice cultivar Zhonghua 11 (ZH11) and then analyzed the F_1_ plants. We found that the heterozygous *Osmtd1* displayed moderate phenotypes in terms of the plant height, tiller number, length of main panicles, and the total and filled grain number (Table 1), indicating that *Osmtd1* was a semi‐dominant mutant. The T‐DNA insert harbor a *bar* resistance gene conferring the transgenic plants resistant to the herbicide phosphinothricin (PPT). We then investigated whether the PPT resistance could be cosegregated with the phenotypes of *Osmtd1*. We generated an F_1_B_1_ population by crossing F_1_ plants to ZH11. The progenies in the F_1_B_1_ population were either PPT‐resistant or PPT‐sensitive. We found that all the PPT‐resistant plants displayed moderate dwarf and multi‐tillering phenotypes, while all the PPT‐sensitive plants had no difference from wild‐type ZH11. We further generated the F_2_ populations. We analyzed the segregation ratio of PPT‐resistant plants to PPT‐sensitive ones using the F_2_ populations and found that the ratio is statistically 3:1 (Table 2). Phenotype analysis showed that all the PPT‐resistant plants in the F_2_ populations displayed strong or moderate phenotypes in plant architecture, while the PPT‐sensitive plants showed no phenotypes (Table 2), indicating that the phenotypes of *Osmtd1* may be cosegregated with PPT‐resistance conferred by T‐DNA insertion. Furthermore, we observed that the ratio of the plants without phenotypes, the ones with moderate phenotypes and the ones with strong phenotypes, is approximately 1:2:1 (Table 2), suggesting that the phenotypes of *Osmtd1* were possibly caused by a single mutated locus.

**Table 1:** The main agronomic traits of rice *Osmtd1*, ZH11, F_1_ hybrids, and F_1_B_1_ hybrids

Lines	Plant height	Effective tillers	Length of main panicles	Total grain numbers	Filled grain numbers
ZH11	104.2 ± 5.2	11.2 ± 1.8	21.6 ± 1.16	165.2 ± 4.21	157.4 ± 3.3
*Osmtd1*	68.6 ± 3.3	28.4 ± 6.6	13.6 ± 0.42	42.2 ± 7.33	41.6 ± 6.66
ZH11/*Osmtd1* F_1_	86.4 ± 4.3	17.7 ± 7.7	18.4 ± 1.49	92.3 ± 18.4	88.9 ± 18.2
*Osmtd1*/ZH11 F_1_	85.5 ± 3.1	19.3 ± 6.7	17.4 ± 1.49	82.5 ± 13.4	78.2 ± 15.2
ZH11/*Osmtd1*/ZH11 R	88.3 ± 2.5	15.3 ± 4.4			
ZH11/*Osmtd1*/ZH11 S	103.7 ± 2.2	8.7 ± 3.4			
*Osmtd1*/ZH11/ZH11 R	89.3 ± 4.0	17.2 ± 6.1	17.8 ± 3.03	103.4 ± 14.7	96.6 ± 11.13
*Osmtd1*/ZH11/ZH11 S	101.2 ± 1.8	6.8 ± 1.1	21.4 ± 0.66	172.8 ± 8.14	162.6 ± 8.65

Data were means ± *SE*. R indicated resistant lines and S indicated sensitive lines to the herbicide phosphinothricin (PPT). ZH11, Zhonghua 11.

**Table 2:** The χ_c_^2^ analysis using F_2_ population from the cross of *Osmtd1* and ZH11

Lines	Numbers	Resistance		Sensitive		χ_c_^2^	Tall		Semidwarf		Dwarf		χ_c_^2^
		Trial	Theoretical	Trial	Theoretical		Trial	Theoretical	Trial	Theoretical	Trial	Theoretical	
*Osmtd1*/ZH11 I	321	245	240.75	76	80.25	0.354	76	80.25	163	160.5	82	80.25	0.313
*Osmtd1*/ZH11 II	131	97	98.25	34	32.75	0.043	34	32.75	63	64.5	34	32.75	0.128
*Osmtd1*/ZH11 III	74	58	55.5	16	18.5	0.631	16	18.5	40	37	18	18.5	0.63
*Osmtd1*/ZH11 IV	165	121	123.75	44	41.25	0.20	44	41.25	86	82.5	35	41.25	1.42
*Osmtd1*/ZH11 Total	691	521	518.25	170	172.75	0.082	170	172.75	352	344.5	169	172.75	0.27
ZH11/*Osmtd1* I	334	245	250.5	89	83.5	0.426	89	83.5	157	167	88	83.5	1.206
ZH11/*Osmtd1* II	142	102	106.5	40	35.5	0.645	40	35.5	69	71	33	35.5	0.817
ZH11/*Osmtd1* III	87	68	65.25	19	21.75	0.629	19	21.75	51	43.5	17	21.75	2.81
ZH11/*Osmtd1* IV	229	166	171.75	63	57.25	0.667	63	57.25	115	114.5	51	57.25	1.28
ZH11/*Osmtd1* Total	792	581	594	211	198	1.053	211	198	392	396	189	198	0.723

### The T‐DNA insertion was located in an unknown gene

**Figure 2:**
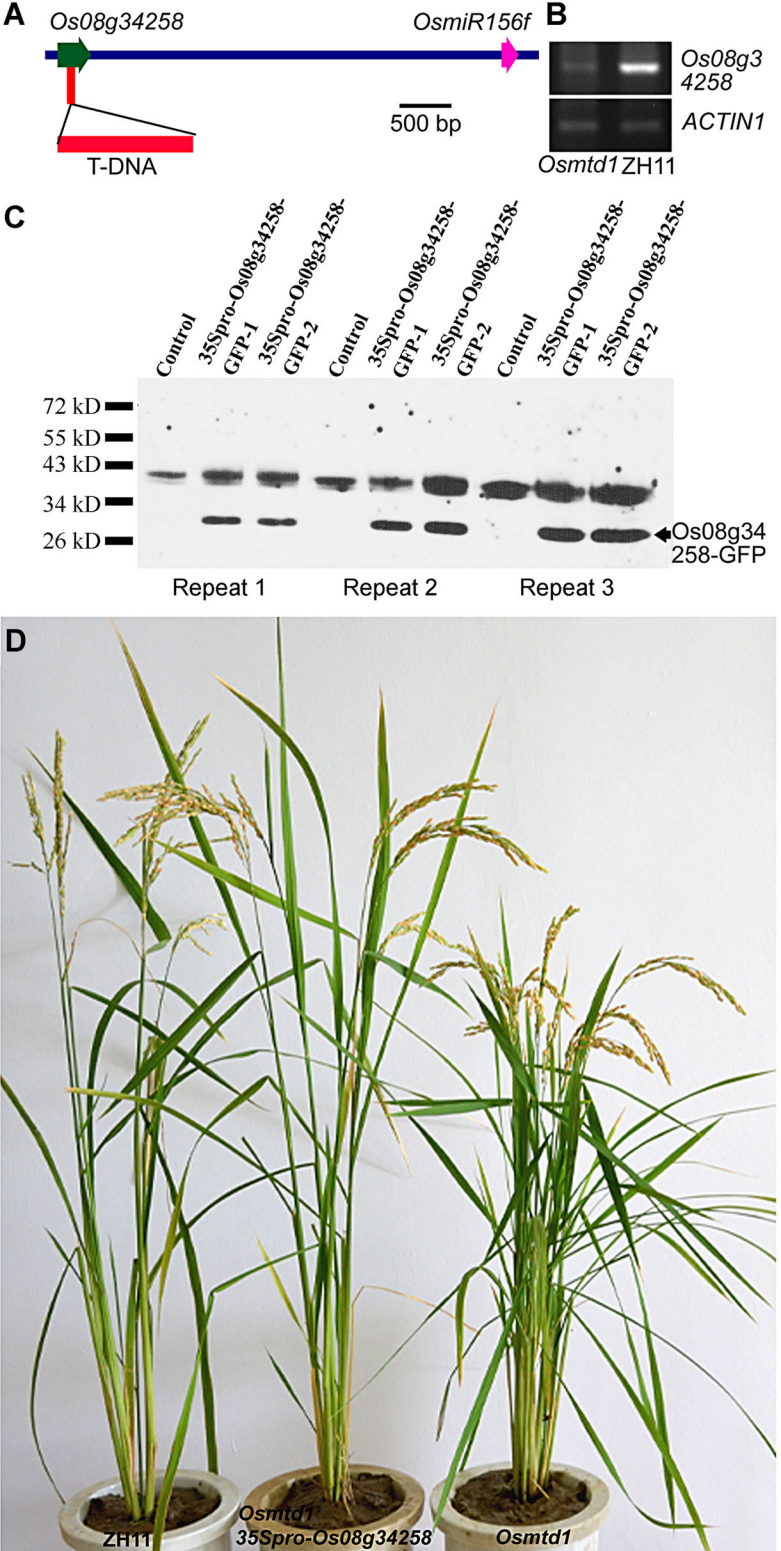
**An unknown gene, *Os08g34258,*was involved in the mutant phenotypes** (**A**) The T‐DNA insertion was located in the middle of a putative gene, *Os08g34258*, which is approximately 3.3 kb upstream of the *OsMIR156f* gene on the eighth chromosome in the *Osmtd1* mutant. (**B**) *Os08g34258* was possibly knocked out in *Osmtd1*. (**C**) Transient expression of Os08g34258 in tobacco leaves showed that *Os08g34258* encodes a protein by western blotting. The controls were the samples from the non‐transformed tobacco leaves. (**D**) Overexpression of the *Os08g34258* gene complemented the phenotypes of *Osmtd1*.

To reveal the molecular base of the phenotypes in *Osmtd1*, we first identified the T‐DNA flanking sequence by thermal asymmetric interlaced polymerase chain reaction (TAIL–PCR) (Liu and Chen 2007). A BLAST search on the Rice Genome Annotation Project website showed that the T‐DNA insertion was localized in the middle of an unknown gene, *Os08g34258*. *Os08g34258* has no introns and *OsmiR156f* was located in the 3.3 kb downstream of it (Figures 2A, S1). A database search showed that *Loc_Os08g34249* had the same sequence as *Os08g34258* in rice genome. Reverse transcription (RT)‐PCR analysis revealed that *Os08g34258* was possibly knocked out in *Osmtd1* because the T‐DNA was inserted in the exon of *Os08g34258* and the trace of product may be derived from *Os08g34249* (Figure 2B). *Os08g34258* encodes a putative protein containing only 67 amino acid residues and a conserved domain shared by the protease inhibitor I family (potato inhibitor type I). This protein family contains a class of small protease inhibitors widely spreading in plants and has been found in potato, tomato, barley, pumpkin, buckwheat, *Solanum nigrum*, and so on (Melville and Ryan 1972; Svendsen et al. 1980; Plunkett et al. 1982; Krishnamoorthi et al. 1990; Hartl et al. 2010; Wang et al. 2011). No functions of this kind of protease inhibitor have been reported in rice. To first test whether *Os08g34258* could encode a protein, we cloned *Os08g34258* and generated the construct 35Spro‐*Os08g34258‐GFP* in which *Os08g34258* was fused with *GFP* and driven by the CaMV 35 S promoter. We transformed 35Spro‐*Os08g34258‐GFP* into tobacco leaves. Western blotting showed that a clear Os08g34258‐GFP band was observed in the isolates from leaves transformed with 35Spro‐*Os08g34258‐GFP* but not in the control, indicating that *Os08g34258* is a real gene (Figure 2C). We then generated the construct 35Spro‐*Os08g34258* in which *Os08g34258* was driven by the CaMV 35 S promoter. We transformed 35Spro‐*Os08g34258* into *Osmtd1* and obtained 31 transgenic rice plants which recovered to the architecture of wild‐type plants (Figure 2D), indicating that disruption of *Os08g34258* by T‐DNA insertion caused the defects of plant architecture in *Osmtd1*. To further demonstrate the roles of *Os08g34258* in rice architecture, we performed RNA interference (RNAi) to knockdown *Os08g34258* in wild‐type ZH11. In the RNAi plants, we indeed observed the dwarfism and multi‐tillering phenotypes similar to those observed in *Osmtd1* (Figure S2), indicating that *Os08g34258* may play important roles in plant architecture.

### *OsmiR156f* was upregulated in *Osmtd1*

**Figure 3:**
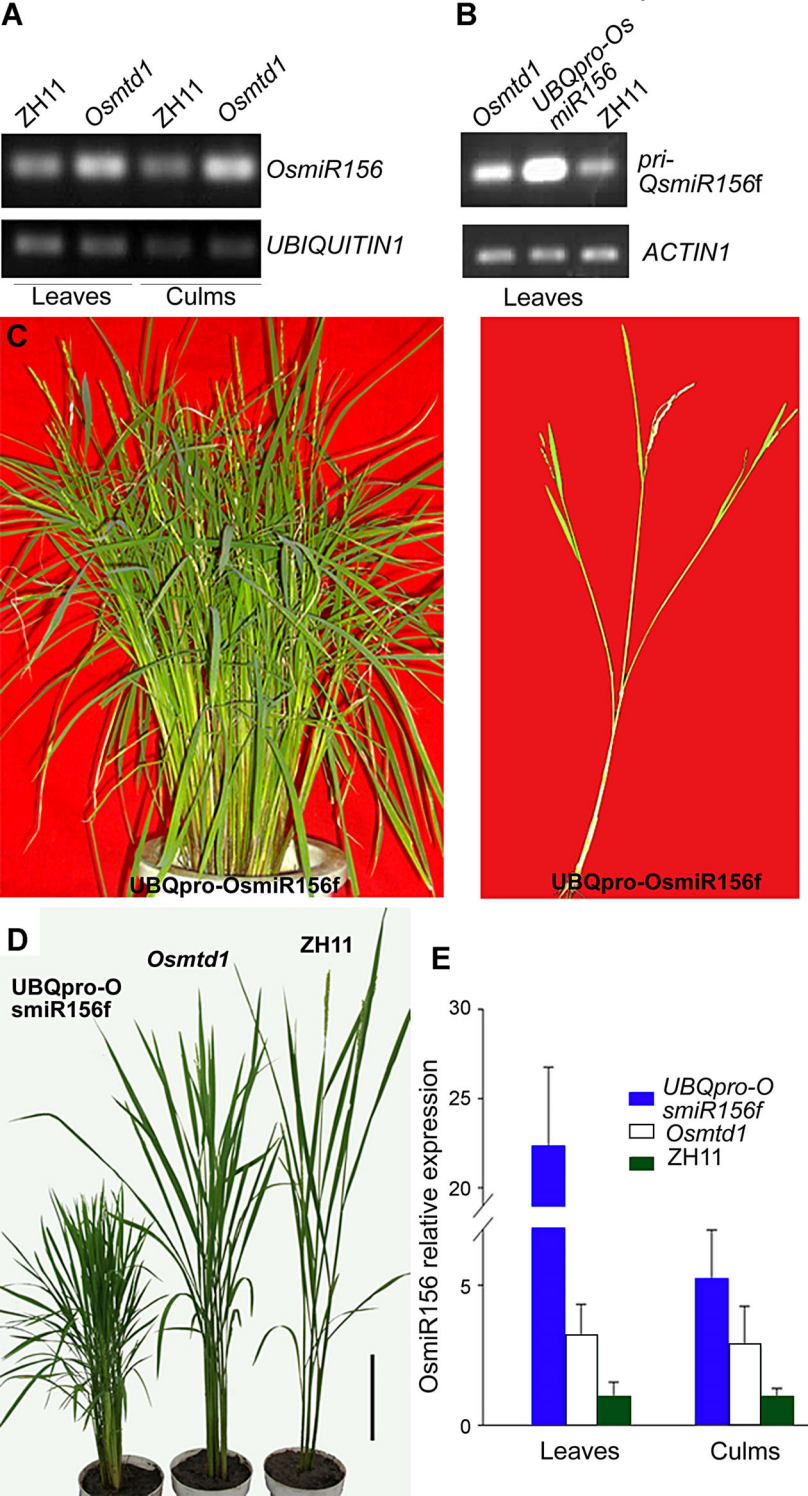
***OsMIR156f* was upregulated in *Osmtd1*** (**A**) *OsmiR156* was upregulated in the leaves or culms of *Osmtd1*. *UBIQUTIN1* was used as the internal control. (**B**) The expression level of *pri‐OsMIR156f* was increased in the leaves of *Osmtd1* or a UBQpro‐OsmiR156f transgenic line. (**C**) A UBQpro‐OsmiR156f transgenic line displayed a bushy phenotype (left) and the outgrowth of axillary tiller in the elongated nodes (right). (**D**) The comparison of phenotypes of a UBQpro‐OsmiR156f transgenic line, *Osmtd1*, and Zhonghua 11 (ZH11). (**E**) The relative expression level of *Os‐miR156* was identified by quantitative reverse transcription polymerase chain reaction in a UBQpro‐OsmiR156f transgenic line, *Osmtd1*, and ZH11.

The *OsmiR156* family gene *OsmiR156d* and *OsmiR156h* play essential roles in plant architecture (Xie et al. 2006). We found that *Osmtd1* displayed similar phenotypes as those observed in the *OsmiR156d* or *OsmiR156h* overexpressing rice plants. Considering that the *Os08g34258* gene is located in the 3.3 kb upstream of *OsmiR156f* (Figure 2A), we hypothesized that *OsmiR156f* may be regulated in *Osmtd1*. To test the hypothesis, we analyzed the expression level of *OsmiR156*. Interestingly, the transcripts of the mature *OsmiR156* were increased significantly in *Osmtd1* (Figure 3A). We further determined that the transcripts of the *OsmiR156f* precursor were increased in *Osmtd1* (Figure 3B), suggesting that the upregulation of *OsmiR156f* may be the molecular base of multi‐tillering and dwarfism in *Osmtd1*.

To further confirm that the excessive production of *OsmiR156f* accounts for the phenotypes of *Osmtd1*, we generated the construct UBQpro‐OsmiR156f in which the *OsmiR156f* precursor was driven by the *UBIQUITIN1* promoter from maize (Cornejo et al. 1993). We transformed UBQpro‐OsmiR156f into ZH11. Among the 217 UBQpro‐OsmiR156f transgenic lines, more than 180 plants displayed dwarfism and multi‐tillering similar to those observed in *Osmtd1*. Some lines showed severe phenotypes with more than 100 tillers (Figure 3C). Similar to *Osmtd1*, the axillary tiller buds in higher internodes were also observed in UBQpro‐OsmiR156f transgenic lines (Figure 3C). We then analyzed the expression level of *OsmiR156f* in the UBQpro‐OsmiR156f transgenic line, *Osmtd1*, or wild‐type ZH11. The results showed that the *OsmiR156f* expression level was highly increased in the UBQpro‐OsmiR156f transgenic line and in *Osmtd1*, when compared with that in wild‐type ZH11. These results demonstrated that *OsmiR156f* was critical for rice tillering and plant height.

### *OsmiR156f* regulated *SQUAMOSA PROMOTER BINDING PROTEIN‐LIKE* (*SPL*) genes at a post‐transcriptional level

**Figure 4:**
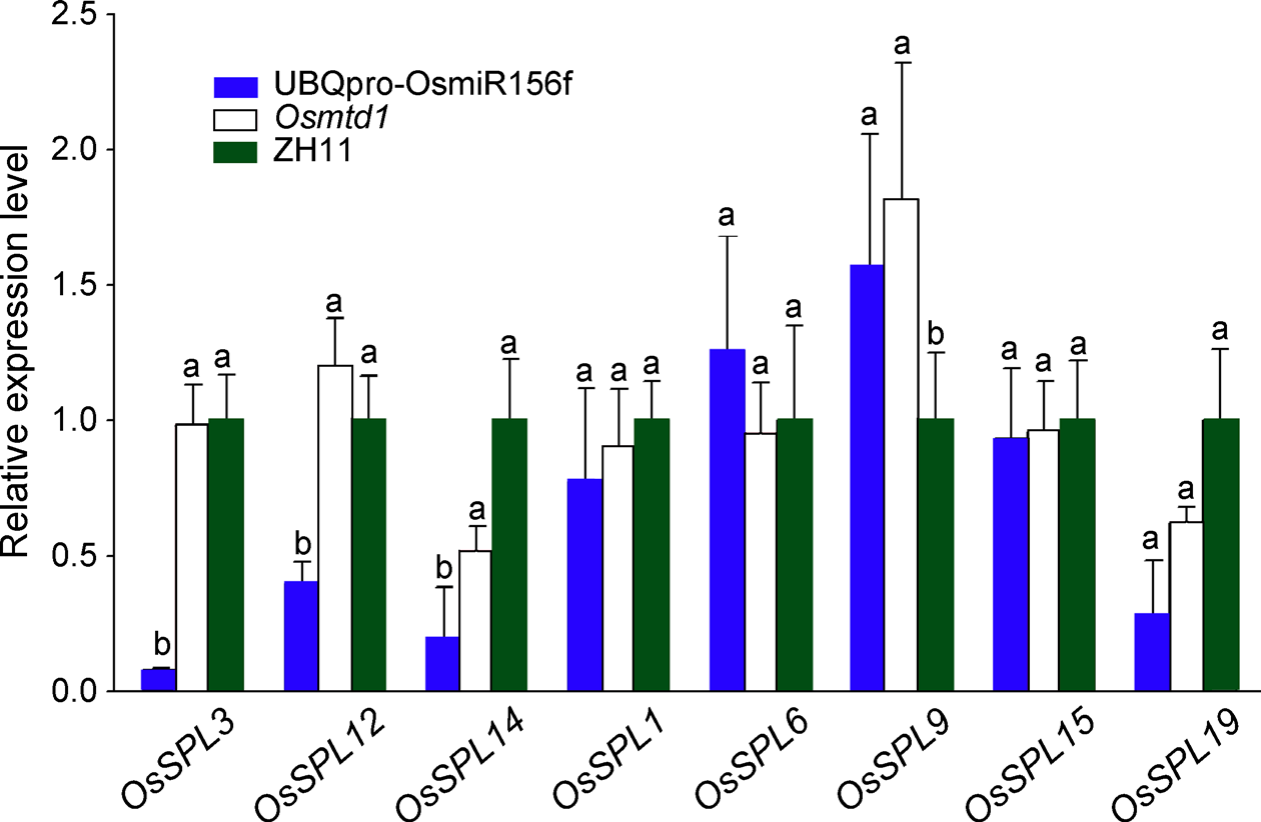
**The regulation of *OsSPLs*by *OsmiR156f*** The expression level of *OsSPL* genes in the young culms from an UBQpro‐OsmiR156f transgenic line, *Osmtd1,* or Zhonghua 11 (ZH11) was revealed by quantitative reverse transcription polymerase chain reaction. The expression levels of the genes in ZH11 were set to 1.0. The error bars represent the standard deviation of three biological replicates.

It has been suggested that *SPL* genes are the direct targets of *miR156* (Rhoades et al. 2002; Xie et al. 2006; Guo et al. 2008). To determine which *OsSPLs* were specifically degraded by *OsmiR156f*, we analyzed the expression level of all the 19 *OsSPLs* in *Osmtd1* and an UBQpro‐OsmiR156f transgenic line by quantitative PCR (qRT–PCR) (Figure 4). The results showed that only eight *SPL* genes (*OsSPL1*, *OsSPL3*, *OsSPL6*, *OsSPL9*, *OsSPL12*, *OsSPL14*, *OsSPL15*, and *OsSPL19*) were detected in young culms. Among them, four *SPL* genes (*OsSPL3*, *OsSPL12*, *OsSPL14*, and *OsSPL19*) were downregulated significantly in UBQpro‐OsmiR156f transgenic plants, two genes (*OsSPL14* and *OsSPL19*) were downregulated obviously in *Osmtd1*, three genes (*OsSPL1*, *OsSPL6*, *OsSPL1*5) had no changes at the expression level in both UBQpro‐OsmiR156f and *Osmtd1*, and one gene *OsSPL9* was unexpectedly upregulated in UBQpro‐OsmiR156f and *Osmtd1*. Among the four genes downregulated in UBQpro‐OsmiR156f or *Osmtd1*, *OsSPL3*, *OsSPL12*, and *OsSPL14* were putative target genes of *OsmiR156* (Xie et al. 2006), suggesting that *OsmiR156f* may specifically regulate *OsSPL3*, *OsSPL12*, and *OsSPL14* in the young culms.

## DISCUSSION

Plant architecture is crucial for yield in rice. The development of IPA has been proved to facilitate rice yield increase (Jiao et al. 2010; Miura et al. 2010). Plant height and tiller number are the two most important agronomic traits for IPA in rice. In this study, we identified a new semi‐dominant rice mutant, *Osmtd1*, that displayed dwarfism, multi‐tillering, late flowering, and shortened panicle size. Molecular analysis suggested that *Os08g34258* encoding a protease inhibitor I family protein and the miRNA gene *OsmiR156f* were affected in *Osmtd1*. Overexpression of *Os08g34258* complemented the phenotypes of *Osmtd1*, while overexpression of *OsmiR156f* in the wild type recapitulated abnormal phenotypes of *Osmtd1*. Our findings established the important roles of *Os08g34258* and *OsmiR156f* in control of plant architecture in rice.

Rice tillering is affected by various factors including the ambient temperature, the plant density, the axil position, and the plant age. Genetic analysis has identified important genes essential for tiller development. The temporal and spatial accumulation of these regulators determined the proper development of rice tillers. *MOC1* initiates axillary buds and promoted tiller outgrowth. *MOC1* encodes a putative GRAS family nuclear protein regulated by 26 S proteasome degradation (Li et al. 2003; Lin et al. 2012; Xu et al. 2012). OsPUP7 has a dominant effect on rice tiller number by regulating the transport of cytokinins (Qi and Xiong 2013). Whereas, *OsTB1* encoding a TCP transcription factor represses axillary bud activity (Takeda et al. 2003). Several factors involved in strigolactone (SL) signaling and biosynthesis have been found to be important for controlling rice architecture. D3 is a homolog of *Arabidopsis* MAX2/ORE9 possessing leucine‐rich repeat (LRR) and F‐box domain and is an important component in SL signaling in control of rice tillering (Ishikawa et al. 2005). D53 functions as a repressor of SL signaling and affects axillary bud outgrowth (Jiang et al. 2013; Zhou et al. 2013). D14 encodes a proposed SL receptor for perception in SL signaling and inhibits rice tillering (Arite et al. 2009; Nakamura et al. 2013). HTD1, D27, and D10 all function in SL biosynthesis and control rice tillering (Zou et al. 2006; Arite et al. 2007; Lin et al. 2009). Some factors including *LAZY1*, *LPA1*, and *PROG1* regulate the angle of rice tillering (Li et al. 2007; Jin et al. 2008; Tan et al. 2008; Wu et al. 2013). In this paper, we identified a new gene, *Os08g34258*, that may also participate in the regulation of rice tillering and plant height. *Os08g34258* encodes a protease inhibitor I family protein which serves as a defensive compound against pathogens. The protease inhibitor inactivates the hydrolytic cleavage ability of proteases which are important pathogenic substances secreted by insects and pathogenic microorganisms. We first discovered the roles of the protease inhibitor I family protein in the development of plant architecture. This protein is completely different from the above‐mentioned known factors which are transcription factors, signaling components, and enzymes in SL biosynthesis, indicating that *Os08g34258* may use a new mechanism to control plant architecture.

The miRNA miR156 family contains 12 members in rice and is highly conserved in plants. Some members of this gene family have been identified to play essential roles in plant development. Constitutive expression of *miR156* homologous genes in different plants causes similar morphological changes including increased number of axillary buds and dwarfism, late flowering, and low fertility (Schwab et al. 2005; Xie et al. 2006; Chuck et al. 2007, 2014). In this paper, we demonstrated that the mutant *Osmtd1* and *OsmiR156f* overexpression lines showed dramatic phenotypes similar to those observed in the overexpression lines of *OsmiR156d* or *OsmiR156h* (Xie et al. 2006). However, we found that *OsSPL3*, *OsSPL12*, and *OsSPL14*, but no other *OsSPLs*, were targeted by *OsmiR156f*, indicating that different *OsmiR156* genes may have differentiated functions in the regulation of rice development. In addition, we unexpectedly found that the expression level of *OsSPL9* was significantly increased in the *OsmiR156f* overexpression lines, suggesting that the regulation between *OsSPL* family genes and *OsmiR156* family may be complicated.

In the *Osmtd1* mutant, the expression level of *Os08g34258* was possibly knocked out, while the expression level of *OsmiR156f* was increased. Interestingly, when we searched the rice database, we found that *Loc_Os08g34249* had the same sequence as *Loc_Os08g34258*. So, it is possible that *Loc_Os08g34249* may also be expressed and may have redundant function with *Loc_Os08g34258*. However, the expression level of *Loc_Os08g34249* is rather low, so it is reasonable that disruption of *Loc_Os08g34258* may lead to obvious phenotypes in rice. The generation of double knockout mutants would be very important for ultimately revealing the function of *Loc_Os08g34258* and *Loc_Os08g34249* in the future. The fact that either knockout of *Os08g34258* or overexpression of *OsmiR156f* led to multi‐tillering and short stature in *Osmtd1* implied that *Os08g34258* may regulate the abundance of *OsmiR156f*, although we cannot provide enough evidence to connect them at present. Especially, *OsmiR156f* is only located in approximately 3.3 kb downstream of *Os08g34258*. It is possible that the T‐DNA insertion may cause both knockout of *Os08g34258* and overexpression of *OsmiR156f*. Further experiments will be needed to verify the possible relationship between *OsmiR156f* and *Os08g34258*. We hypothesize that two mechanisms may exist if *OsmiR156f* were regulated by *Os08g34258* during rice tillering. First, *Os08g34258* may control the *OsmiR156f* transcriptions by indirectly regulating unknown transcription factors which control the transcripts of *OsmiR156f*. Second, as *Os08g34258* encodes a putative protease inhibitor, it may regulate the *OsmiR156f* abundance by negatively affecting the process of miRNA biogenesis. In plants, there are at least two important and critical steps involved in cleavage or dicer processing during miRNA biogenesis: the processing of pri‐miRNAs to pre‐miRNAs, and further processing to miRNA‐miRNA* duplex by DCL1 and its interacting partners (Kurihara and Watanabe 2004; Qi et al. 2005; Kurihara et al. 2006; Chen 2008). Based on the fact that both protease and DCL1 perform a similar role in cleavage of their targets, the protease inhibitor encoded by *Os08g34258* may affect the activities of DCL1 or other enzymes during miRNA biogenesis. The investigation of the interactions between Os08g34258 and the components in the miRNA pathway including DCL1, HYL1 (Kurihara et al. 2006), SE (Lobbes et al. 2006; Yang et al. 2006), HEN1 (Yu et al. 2005), AGO (Baumberger and Baulcombe 2005; Qi et al. 2005, 2006), and HASTY (Bollman et al. 2003; Park et al. 2005) would provide further evidence to prove the possible implications of Os08g34258 in regulation of miRNA biogenesis in the future.

## MATERIALS AND METHODS

### Plant materials

A multi‐tillering and dwarf rice mutant *Osmtd1* was obtained by a T‐DNA insertion mutant collection. The plasmid including maize *Ds* sequence and a *bar* gene (Wang et al. 2000) was induced in *Oryza Sativa* spp. *japonica* cv. ZH11. Agronomic traits were investigated in detail between *Osmtd1* and ZH11 at different developmental stages. Genetic analysis was carried out using *Osmtd1* or ZH11 as pollen donors to obtain F_1_ hybrids of *Osmtd1* × ZH11 or ZH11 × *Osmtd1*. Reciprocal crossing experiments were carried out between F_1_ hybrids and *Osmtd1* or ZH11. The agronomic traits of plant height, tiller number, and panicle length were examined in different hybrid populations.

### Constructs and transformation

To generate 35Spro‐*Os08g34258* in which the *Os08g34258* gene was driven by the CaMV 35 S promoter, the coding region of *Os08g34258* was amplified from spp. *japonica* (cv. *Nipponbare*) genomic DNA using primers 5′‐ATG AGC CAG AAG TCG TGG C‐3′ and 5′‐ACA CAT GAA CGT ACA CGG CGC C‐3′. The fragment was cloned into the *Eco*R V site of pBluescript SK^+^ (pBS) (designated pBS‐inh) and was sequenced. The overexpression construct of 35Spro‐*Os08g34258* was generated by ligation of the *Kpn* I/*Xba* I digested fragment from pBS‐inh and vector pQG111 digested by the same restriction enzymes (Tao et al. 2013). 35Spro‐*Os08g34258* was then transformed into the mutant *Osmtd1*.

To generate 35Spro‐*Os08g34258‐GFP*, the coding region of *Os08g34258* was amplified using the primers 5′‐*CAC C*AT GAG CCA GAA GTC GTC G‐3′ and 5′‐ACC GAT GAC GGG AAT TTT GA‐3′. The fragment was cloned into the vector pENTR/D‐TOPO (Invitrogen, San Diego, CA, USA) to generate pENTRY‐Os08g34258. The 35Spro‐*Os08g34258‐GFP* was generated by LR reaction between pENTRY‐Os08g34258 and pK7FWG2. 35Spro‐*Os08g34258‐GFP* was infiltrated into the leaves of tobacco by *Agrobaterium tumefaciens*‐mediated transformation. The tobacco plants were grown in the dark for 24 h and then in a greenhouse under normal conditions for 48 h. Protein isolates were mixed with sample buffer and separated in 12% sodium dodecylsulfate polyacrylamide gel electrophoresis minigel and then were blotted onto nitrocellulose membranes for western blotting.

To generate UBQpro‐Os08g34258‐RNAi to knockdown *Os08g34258* in ZH11, the fragment was amplified from pBS‐inh plasmid DNA using the primers 5′‐ATG AGC CAG AAG TCG TGG C‐3′ and 5′‐ACG GAC GCG CTT GTC GTT GA‐3′. The fragment was cloned into the *Eco*R V site of pBluescript SK^+^ to obtain the sense insert named as pBS‐△inh and antisense insert named pBS‐A△inh. The intron‐containing construct pBS‐A△inh‐GUS‐△inh was first generated by ligating four fragments including pBS vector digested by *Cla* I/*Pst* I, A△inh fragment from pBS‐A△inh by *Hind* III/*Pst* I digestion, the 1 kb fragment *GUS* fragment from pBS‐GUS by *Eco*R I/*Hind* III digestion, and △inh fragment from pBS‐△inh *Cla* I/*Eco*R I. The UBQpro‐Os08g34258‐RNAi were generated by ligating the pWM101 vector digested by *Hind* III/*Sal* I, the ubiquitin promoter from pBS‐pUBQ with *Hind* III/*Bam*H I digestion, and A△inh‐GUS‐△inh fragment from pBS‐A△inh‐GUS‐△inh digested by *Sal* I/*Bam*H.

To generate UBQpro‐*OsmiR156f* in which the *OsmiR156f* gene was driven by a *UBIQUITIN1* promoter, the *OsmiR156f* precursor was amplified from the *Nipponbare* genomic DNA using primers 5′‐CGC CCA CCT TTC TCC CA‐3′ and 5′‐AAG GAG CAG TTA GAT AAT GGA G‐3′. The fragment was cloned into the *Eco*R V site of pBS (designated pBS‐miR156) and sequenced. The Ubiquitin promoter was released by digesting the pBS‐pUbq construct (Wei et al. 2013) with *Hind* III/*Bam*H I. The pBS‐miR156 was digested by *Sal* I/*Bam*H I, and the fragments including *OsmiR156f*, the pWM101 vector digested by *Hind* III/*Sal* I, and the Ubiquitin promoter released from pBS‐pUBQ with *Hind* III/*Bam*H I digestion were ligated to obtain UBQpro‐*OsmiR156f*. UBQpro‐Os08g34258‐RNAi and UBQpro‐*OsmiR156f* were then transformed into ZH11. The *A. tumefaciens*‐mediated transformation was employed as previously reported (Liu et al. 1998).

### Polymerase chain reaction analysis

The T‐DNA flanking sequence in *Osmtd1* was isolated by TAIL‐PCR method (Liu and Chen 2007). Three specific primers (5′‐CGA TTA CCG TAT TTA TCC CGT TCG‐3′, 5′‐GTT ACC GGT ATA TCC CGT TTT CG‐3′, and 5′‐GAG GTA TTT TAC CGA CCG TTA CCG‐3′) complementary to the *Ds* sequence were used as described (Liu et al. 2007). The T‐DNA insertion site was obtained by searching the T‐DNA flanking sequence in the rice genome using BLAST (Basic Local Alignment Search Tool) software.

To perform RT‐PCR, qRT‐PCR, and Stem‐loop RT‐PCR, total RNA was isolated using Trizol reagent (Invitrogen) and removed DNA contamination with RNase‐free DNase I (Invitrogen). M‐MLV kit (Invitrogen) was used according to the manufacturer's instructions to synthesize first‐strand cDNA for PCR.

Reverse transcription PCR was performed using the 2 × Taq Master Mix (CWbio), and normalized using *ACTIN1* as a standard. Quantitative RT‐PCR experiments were performed using Power SYBRGreen Master Mix (QIAGEN, Hilden, Germany) and 7300 qRT‐PCR System (Applied Biosystems, Foster City, CA, USA) according to the manufacturers’ protocols. *UBQUITIN1* expression was used as an internal control. Three biological replications were analyzed as described previously (Xie et al. 2006). Stem‐loop RT–PCR was employed to detect *Os‐miR156* abundance as reported previously (Varkonyi‐Gasic et al. 2007). The primers used for testing *Os08g34258* expression were 5′‐ATG AGC CAG AAG TCG TCG TGG C‐3′ starting from the ATG codon and 5′‐ACG GAC GCG CTT GTC GTT GA‐3′ at the position 130 bp from the ATG codon. The primers for testing pri‐OsmiR156f were 5′‐CTT CCC TTC GAC AGG ATA GC‐3′ and 5′‐AGC GGC AGC TGT ATC ATC A‐3′. The primers used for different *OsSPL*s and internal control genes are as follows: *OsSPL1*, 5′‐ACC GAA AAG CAC GCG AGC CA‐3′, 5′‐TGG GGT GGC CCC TGC ATG AT‐3′; *OsSPL3*, 5′‐CTG CCG GTG TTG ATG GGA CGC‐3′, 5′‐GCA GAA GCT TCC ATG CCT TGG T‐3′; *OsSPL6*, 5′‐GCT ACA TGC TGC CGT GCG GA‐3′, 5′‐CCG TCT CGC GCG TTT CTC CA‐3′; *OsSPL9*, 5′‐TGC GTC TTG CCA CCA CTG TGG‐3′, 5′‐TTA CTG GCG CGG CAA GCA CC‐3′; *OsSPL12*, 5′‐CCG TCC ACC GGA GGA GGA GG‐3′, 5′‐AGC CGG AGC TGC TGC CAT TG‐3′; *OsSPL14*, 5′‐CCG GTG TTC GCT GGC CCA AA‐3′, 5′‐CAT GGC GGA GGG TGC CAC AG‐3′; *OsSPL15*, 5′‐GTT CGG TCT GGA TCG CCT GGA‐3′, 5′‐CGT TGG TCA GAT CCG CCC TGC‐3′; *OsSPL19*, 5′‐TCA GGG ACC TTC CGG GAA CCG‐3′, 5′‐TGC GCT GCG GGT TGT GGT AA‐3′; *ACTIN1*, 5′‐TGC TAT GTA CGT CGC CAT CCA G‐3′, 5′‐AAT GAG TAA CCA CGC TCC GTC A‐3′; and *UBIQUITIN1*, 5′‐GTG GCC AGT AAG TCC TCA GC‐3′, 5′‐ACA ATG AAA CGG GAC ACG AC‐3′.

## Supporting information

Additional supporting information may be found in the online version of this article at the publisher's web‐site.

**Figure S1.** The T‐DNA flanking sequence in *Osmtd1* mutant

**Figure S2.** RNA interference (RNAi) showed that disruption of *Os08g34258* caused dwarfism and multi‐tillering phenotypes similar to those observed in *Osmtd1*.
