# Supplementary figures and images for: The alteration in the architecture of a T‐DNA insertion rice mutant osmtd1 is caused by up‐regulation of MicroRNA156f
Source: J Integr Plant Biol. 2015 Apr 10;57(10):819–29. doi: 10.1111/jipb.12340 (PMC6681133; doi:10.1111/jipb.12340)

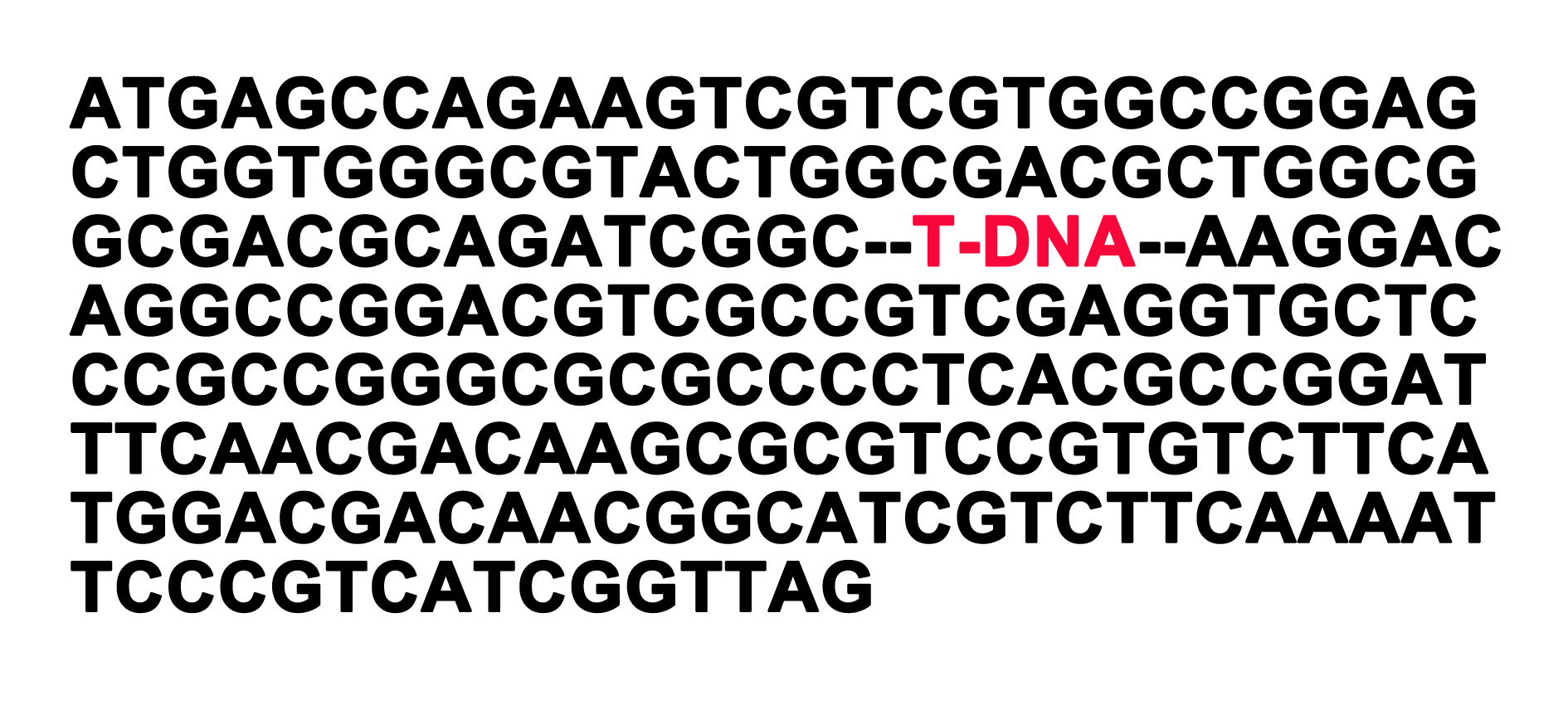

Supplement: Supplementary file 1 — Figure S1. The T‐DNA flanking sequence in Osmtd1 mutant [file JIPB-57-819-s001.tif]

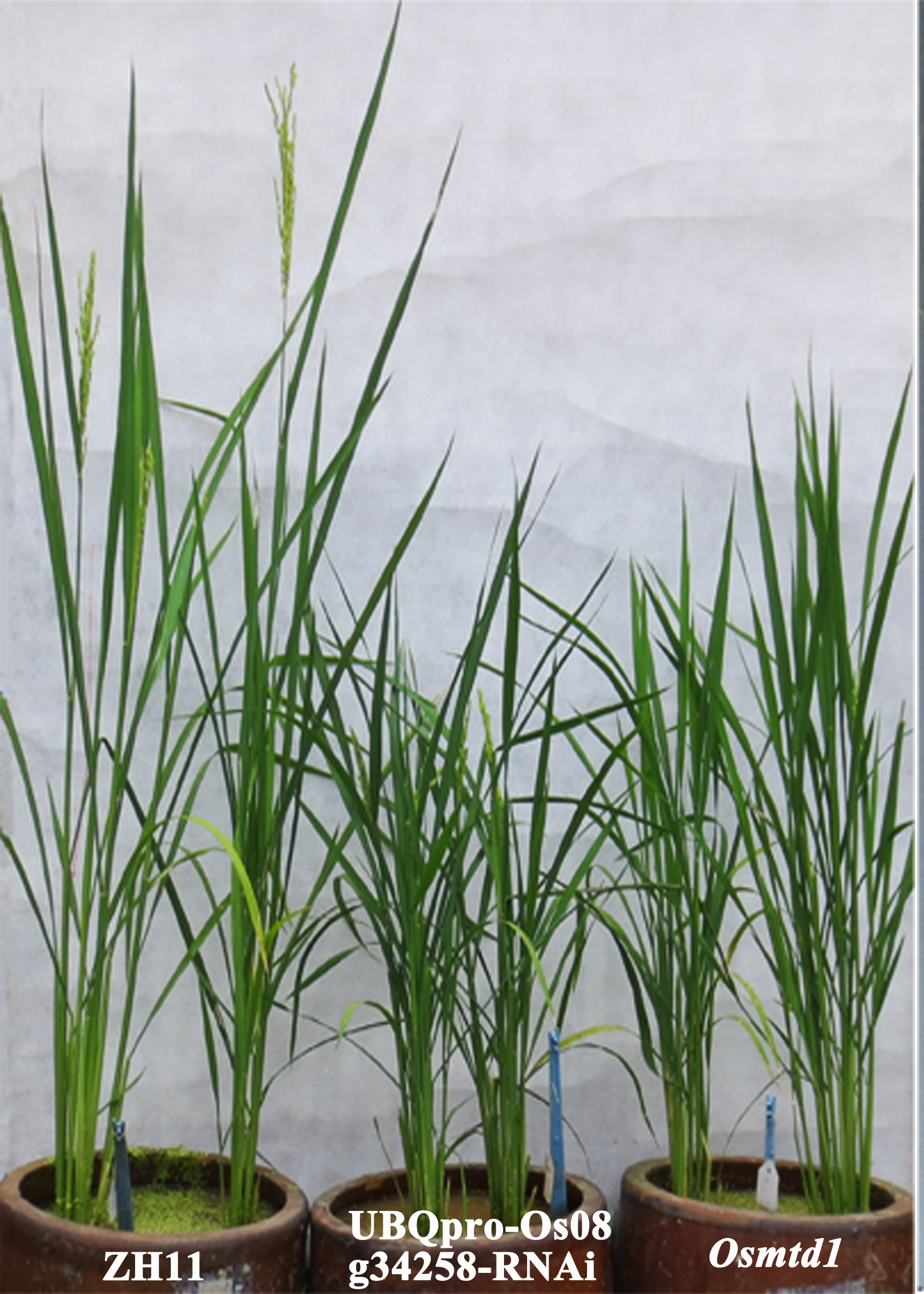

Supplement: Supplementary file 2 — Figure S2. RNA interference (RNAi) showed that disruption of Os08g34258 caused dwarfism and multi‐tillering phenotypes similar to those observed in Osmtd1. [file JIPB-57-819-s002.tif]
